# Implicit task switching in Parkinson’s disease is preserved when on medication

**DOI:** 10.1371/journal.pone.0227555

**Published:** 2020-01-14

**Authors:** Jacob A. Yaffe, Yair Zlotnik, Gal Ifergane, Shelly Levy-Tzedek

**Affiliations:** 1 Goldman Medical School, Faculty of Health Sciences, Ben-Gurion University of the Negev, Beer-Sheva, Israel; 2 Neurology Department, Soroka University Medical Center, Beer-Sheva, Israel; 3 Recanati School for Community Health Professions, Department of Physical Therapy, Ben-Gurion University of the Negev, Beer-Sheva, Israel; 4 Zlotowski Center for Neuroscience, Ben-Gurion University of the Negev, Beer-Sheva, Israel; 5 Freiburg Institute for Advanced Studies (FRIAS), University of Freiburg, Freiburg, Germany; University College London, UNITED KINGDOM

## Abstract

People with Parkinson’s disease have been shown to have difficulty switching between movement plans. In the great majority of studies, the need to switch between tasks was made explicitly. Here, we tested whether people with Parkinson’s disease, taking their normal medication, have difficulty switching between implicitly specified tasks. We further examined whether this switch is performed predictively or reactively. Twenty five people with Parkinson’s disease continuously increased or decreased the frequency of their arm movements, inducing an abrupt–but unaware–switch between rhythmic movements (at high frequencies) and discrete movements (at low frequencies). We tested whether that precipitous change was performed reactively or predictively. We found that 56% of participants predictively switched between the two movement types. The ability of people with Parkinson’s disease, taking their regular medication, to predictively control their movements on implicit tasks is thus preserved.

## Introduction

Parkinson’s disease (PD) is a progressive neurodegenerative disease [1]. Considered the second most common neurodegenerative disease after Alzheimer’s disease, PD effects approximately 7.5 million people worldwide [2]. The prevalence of PD differs with age, and affects 19 people out of 1000 in people 80 years and older [2]. PD is diagnosed based on the presence of Parkinsonism, a clinical manifestation which is defined as bradykinesia (slowness of movement) in combination with either rest tremor, rigidity or both [3]. Slowness of movement and prolonged reaction times [4] affect many aspects of daily life such as speech, writing, facial expressions, rising from a chair, walking, and automatic movements, like reduced arm swing when walking [5]. Freezing of Gait (FOG) is characterized by sudden and brief episodes of start hesitation or inability to move the feet, usually during gait initiation or turning [4,6], and is considered a major risk factor for falls in PD [7]. FOG is often manifest when patients have to switch their movement plan: e.g., when trying to negotiate an obstacle while walking [7]. The physical examination of these cardinal manifestations is based on the Motor Examination Section of the MDS-UPDRS [8].

The ability to respond to changes in the surrounding is of high importance in everyday life. When one’s immediate environment calls for a change in behavior–e.g., a puddle on the path ahead calls for a detour–the response can be to either *pre-act* [9], and take the detour before reaching the puddle, or *re-act*, and take it once one’s shoes are wet and muddy. Pre-action can help prevent injuries or even avoid life-threatening situations [10,11]. It has been documented that people with PD have difficulties switching between movement plans [12], such as walking initiation and turning [13,14], or trying to perform a predetermined sequence of actions [15], and that transitioning between tasks (e.g., rising from a chair) is associated with an increased risk of falling [7]. And so, it is of prime importance to understand the factors that affect poor transitioning between movement plans in PD. In the tasks reviewed above, as well as others documenting a difficulty for patients with PD in switching between tasks [16–19], the need to change one’s movement plan is explicitly clear. But does this difficulty in switching persist when the requirement to switch is implicit?

Implicit sensory cues are perceived throughout everyday activities, and integrated to form appropriate responses to changes in one’s surroundings. Examples of such implicit control of movement have been recorded in object manipulation tasks [20], showing grip force variations [21,22], in collaborative tasks–like the mirror game [23,24], and in postural control. While coordinating the activity of muscles to maintain the body’s balance is a voluntary control process, it is achieved without being explicitly aware of the constant updating of the movement plans needed. Anticipatory postural adjustments (APAs) are made when people are aware that they will have to adjust their balance, e.g., because they are about to lift a heavy object [25,26]. It has been shown that, in those cases, the explicit portion of the movement (e.g., reaching to a high shelf to obtain a heavy object) is controlled separately from the implicit portion of the movement–the APAs, which stabilize the body during the reaching and lifting motion [25]. People with PD have been shown to have APA durations similar to those of healthy individuals [27,28], which may have suggested that implicit control of full-body movement is not impaired in people with PD. However, the APA durations of people with PD have been found to be more variable [27,29], and multiple APAs, rather than a single one, were recorded in people with PD who exhibit FOG [30]. The study of APAs is concerned with lower-limb adjustment to anticipated change in posture. Here, we set out to test whether people with PD have difficulties switching between implicitly specified tasks of the upper limb.

Our goals in the current study were to test: (1) whether patients with PD, taking their regular medication, are able to switch between two movement plans when the requirement to switch is implicit; And if so, (2) whether the switch occurs pre-emptively, or reactively.

**Fig 1:**
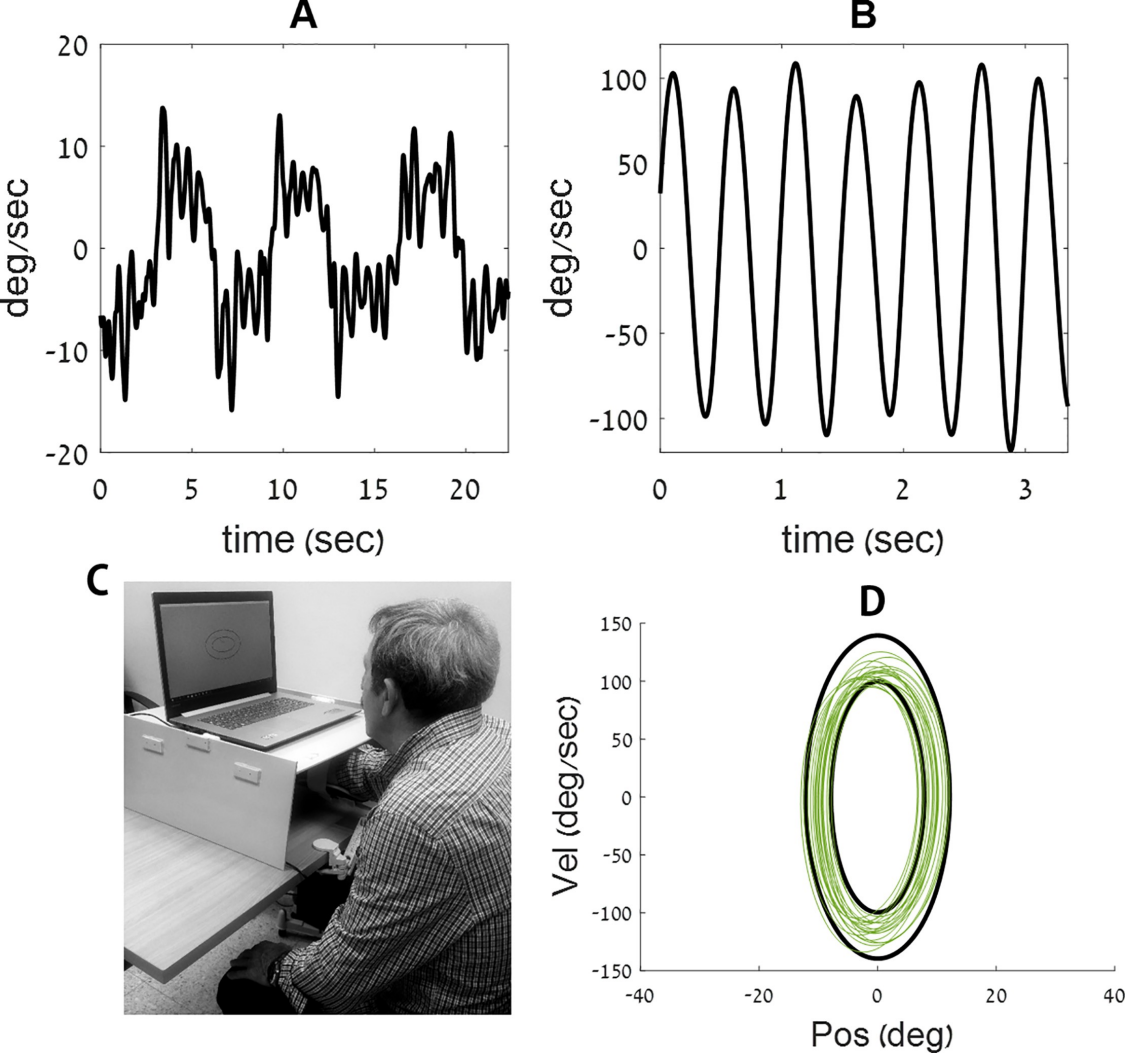
Examples of velocity traces, from the current experiment, recorded during a discrete (A) and during a rhythmic (B) movement performed by participant #1. (C) The participant places his arm on the armrest, below an opaque cover. He uses the movements of his forearm to control a cursor on a phase plane, displayed on a computer screen (D) example of a training ellipse ‘C’ from the current experiment, shown in black on a phase plane, along with the movement trace of participant # 1 (in green).

We previously identified two distinct movement types–rhythmic and discrete [31–33] when individuals make repetitive movements with their forearm (see Fig 1A and 1B, respectively) [33,34]. This distinction is similar to that made between running and walking. Having shown that individuals perform rhythmic movements at high forearm frequencies, and discrete movements at low forearm frequencies, we set out to test the nature of the transition between these two movement types–rhythmic and discrete.

In these studies, we asked participants to perform out-and-back movements with their forearm parallel to the ground at continuously increasing or decreasing frequencies, and studied the transitions between the two movement types [9,32]. We found that the *gradual* change in movement frequency induced an unaware *abrupt* change from a discrete to a rhythmic movement type, and vice versa.

Each individual has a range of arm frequencies at which they can perform both discrete and rhythmic movements, and that range can vary across individuals. We previously showed that switching between discrete and rhythmic movements (and vice versa) was done in an *anticipatory* manner within this range of frequencies that enabled both movement types. In half of the trials the participants were asked to continuously increase the frequency of their arm movements (INC), and in the other half, they were asked to continuously decrease their movement frequency (DEC). We found that the switch frequency was not the same for the INC and the DEC trials. On DEC trials the switch occurred at a *higher* frequency than the frequency on INC trials.

We use the term hysteresis to describe the switching pattern between discrete and rhythmic movements. Hysteresis is the dependence of the state of a system on its history [35]. In this experiment, the hysteresis is the result of switching between movement plans at different frequencies: the switch when the required movement frequency gradually increases occurs at one frequency (FsINC), and when the frequency gradually decreases it occurs at another frequency (FsDEC). A Positive Hysteresis (PH) occurs when FsINC is higher than FsDEC (reactive behavior); a Reverse (or Negative) Hysteresis (RH) occurs when FsINC is lower than FsDEC (predictive behavior).

Had participants maintained the type of movement with which they started (either discrete or rhythmic) for as long as possible before switching to the other movement type, a “classical hysteresis” (or positive hysteresis) (PH) pattern would have emerged. However, in practice, a “reverse hysteresis” (RH) pattern often emerged in their movement data [32].This reverse-hysteresis pattern was interpreted to indicate that participants employed predictive control of movement [9,32].

In a previous study, we found that both aging and cognitive load impaired the ability to predictively control the implicit change between movement types [9]. Would PD have a similar effect? Impairment of motor skills in PD such as the reduced ability to automatically perform tasks [36] and the deficit in task switching [16] informed our working hypothesis that PD impairs predictive control and affects the ability to switch between implicitly specified motor tasks. Specifically, we hypothesized that: (1) the majority of patients with PD will not be able to switch between the two motor tasks; and (2) that patients with PD who will switch between motor tasks, will not do so in a predictive manner (i.e., their FsINC will have a higher value than their FsDEC). Since task-switching difficulties have been recorded both when patients are on [17,37,38] and off [39] medication, we tested patients in the on-medication state.

We have previously shown that people with PD are able to perform a *static* version of the proposed task [40,41], where the required frequency remained constant, and did not dynamically change during a trial (see Methods). Here, participants were asked to continuously change the frequency of their arm movements.

## Methods

### Participants

A total of 29 people with PD were recruited, and of those, 25 completed the experiment (67±8.4 years old, mean±SD; 7 females, 18 males). All patients were tested using their dominant hand (23 right handed, 2 left handed). The exclusion criteria for this experiment were: presence of other parkinsonian disorders; orthopedic problems of the shoulder or elbow joint; and uncorrected vision disorders. All participants gave their written informed consent to participate.

### Equipment

An arm rest, mounted on a table, and connected to an encoder that records the angle of the elbow with an accuracy of 0.002 degrees per count at 200 Hz, was used, as described in [9,32–34,40–43] was used. The arm rest was placed under an opaque cover, so participants could not directly see their arm moving.

### Protocol

At the beginning of the experiment patients were asked to provide details related to the disease onset, PD and hand dominance, sleep habits and PD medication taken. Where appropriate, their medical charts were later consulted to corroborate the details. In order to assess the severity of the patients’ disease, the motor examination section of the MDS-UPDRS [8] was conducted. To assess cognitive skills, the Mini-Mental State Examination [44] was conducted.

Patients were fitted with a wrist brace, to prevent movements of the wrist and were seated in front of a computer screen with their arm placed on the custom-built arm rest. They were asked to move their forearm towards and away from their body in a movement similar to that of a windshield wiper. These were one-dimensional movements, in the horizontal plane. A cursor on the computer screen indicated to participants what their arm's location and speed were at any given time (see Fig 1C), by showing a phase plane: position was indicated on the X axis, and velocity on the Y axis (see Fig 1D). A pair of black concentric ellipses formed a doughnut shape, which defined the amplitude (on the X axis) and speed (on the Y axis) allowed on the task. The participants’ forearm movements controlled the computer cursor, which they were asked to keep within the doughnut-shaped area during each trial (see green trace in Fig 1D). The size of the concentric ellipses on the phase plane thus indicated to the participants the required amplitude, speed and frequency of the movement.

#### Training

Participants were given the opportunity to practice moving their arm so as to keep the cursor within the two concentric ellipses. They were given three different ellipse sizes to practice with, and were explicitly instructed how to keep the cursor between the two ellipses. It was explained to them that their right-left arm movements controlled the right-left location of the cursor, and that the speed of their movement controlled the height of the cursor on the screen.

During the training session, the size of the ellipses did not change during each trial. The ellipses had central frequencies of 0.1Hz, 0.7Hz and 2Hz. Each training trial lasted 40 sec, and participants could repeat these until they felt comfortable with the task.

#### Testing

In trials of the testing session, the height of the ellipses changed dynamically–either increasing, which required increasing the movement speed and frequency, or decreasing, which required a slower, lower-frequency movement. This continuous change in forearm frequency was designed to induce a precipitous switch between the two distinct movement types: *discrete* (at low frequencies) and *rhythmic* (at high frequencies) [32]. Participants were *explicitly* aware of the changing size of the target zone on the screen, which required them to adjust their movement frequency accordingly. However, they were not aware of the switch between the two movement types (rhythmic and discrete) that is imperative for the successful completion of the task. Thus the switch is performed *implicitly*.

The task was chosen to be one that is not familiar from everyday life, so that prior experience would not affect task performance.

**Fig 2:**
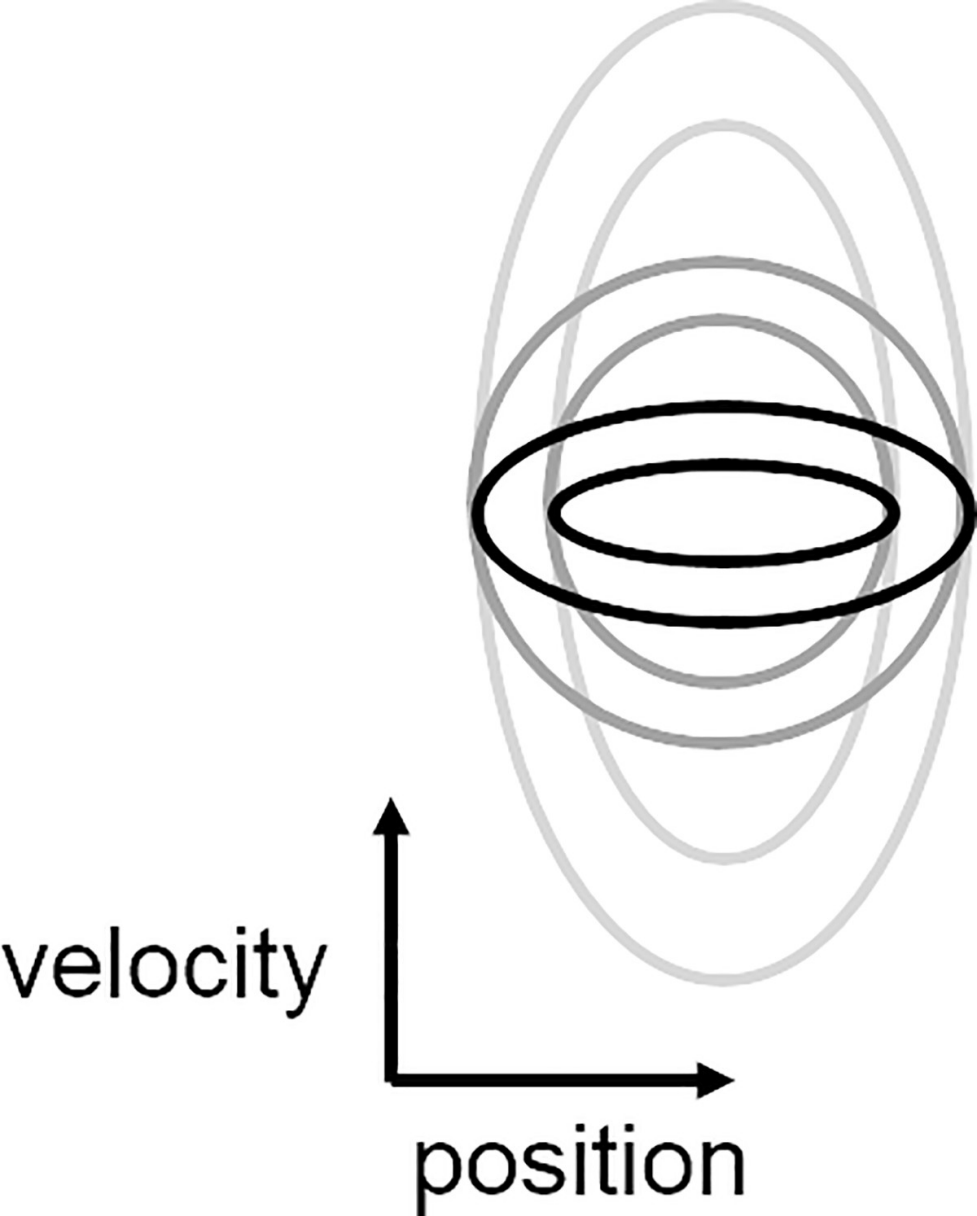
Testing ellipses. The ellipse size was continuously changed during the testing session: the required amplitude (x-axis) remained constant, while the required speed gradually increased or decreased. Shown in black is the ellipse that requires low-frequency forearm movement, and in light-gray is the ellipse that requires high-frequency movement.

The testing session consisted of two blocks of four 66.5-sec trials each. The difference between the two blocks was the direction of the change in frequency, with one block consisting of four increasing-frequency trials (INC) and one with four decreasing-frequency trials (DEC). The smallest ellipse size in the testing session corresponded to the smallest ellipse size in the training session (central frequency = 0.1 Hz), and the same was the case for the largest ellipse size (central frequency = 2 Hz). Fig 2 illustrates the changes in ellipse height during the testing session. The required arm amplitude did not change during the entire experiment, and was set at 20±3º. The order at which participants performed the two blocks (INC/DEC) was counter-balanced.

Ethical approval for this study was obtained from the Helsinki Committee at the Soroka hospital, where the study was conducted.

### Data analysis

The angle of the elbow, was filtered with a 1^st^- order Butterworth filter (cutoff frequency of 20 Hz). To reduce the effects of drift, the best straight-line fit from the position data was removed. The MATLAB^®^ software (Mathworks, MA, v.9.3) was used for data analysis. The movement type performed by participants at every time point was determined using the **harmonicity** index [9,31–33,45–47], a unitless number that is calculated for each movement half-cycle (each flexion or extension movement is considered a half cycle). A harmonicity value equal, or close to 1, indicates a highly rhythmic movement. It is calculated as follows: when there is a single peak in acceleration during the movement, the harmonicity index is assigned the value of one; if there are multiple peaks in the acceleration, the harmonicity index is assigned the value of the ratio of the minimum to the maximum absolute values of the acceleration within the given movement; If there is a change in the sign of the acceleration during the movement, the harmonicity index is assigned the value of zero [45,46]. This index has been shown to be a robust indicator of movement type (see, for example, [33]).

The harmonicity values were used to determine the frequency (**Fs**) at which a switch occurred between movement types [9,32]. In INC trials, the first instance of four consecutive harmonicity values greater than 0.5 was identified as the switch point between the discrete and the rhythmic movement types. The value of 0.5 was chosen to comply with convention [9,32,43,46–49]. A trial was considered a no-switch (NS) trial if it did not contain four consecutive half-cycles with a harmonicity value greater than 0.5.

Similarly, in DEC trials, the first instance of four consecutive harmonicity values *smaller* than 0.5 was identified as the switch point between the rhythmic and the discrete movement types. A DEC trial was considered a no-switch trial if it did not contain four consecutive half-cycles with a harmonicity value smaller than 0.5.

Validation of this method for the detection of the switch frequency had been done using a sigmoid function fit [32].

#### Switch frequency and predictive control

For each participant, we identified the frequency at which they switched between the rhythmic and discrete movement types (FsDEC, the switch frequency on DEC trials), and between discrete and rhythmic movement types (FsINC, the switch frequency on INC trials). We tested whether there was a switch between movement types (discrete and rhythmic) at all, and, if there was one, whether the participants performed the switch in a predictive manner (that is, whether FsDEC was higher than FsINC).

#### Statistical analysis

We used the Wilcoxon signed rank test for paired observations to compare the FsINC and FsDEC values. We employed a conservative approach: in cases where there was only one INC or DEC trial with a switch point, out of the four trials in that block, and an average between two or more values could not be taken, this participant’s data were not included in the statistical analysis, to avoid bias due to sampling error.

## Results

Twenty five participants completed the experimental procedure (see Table 1; see S1 Table for rigidity scores per participant). Of those, one participant completed only three INC trials, rather than four, due to a technical error (Patient #3). Three participants (#3, #8 and #18) reported past or present impairment in the shoulder joint (e.g. pain or injury), and five participants (#5, #13, #16, #18 and #19) reported past or present backache (e.g. pain, lumbar disc herniation or lumbar disc stenosis). Eleven participants reported having sleep disorders. All participants reported taking their PD medications except one participant (#3) who preferred avoiding medications at that stage of his disease. Medication dosages per participant are reported in S2 Table. All participants reported normal or corrected-to-normal vision.

**Table 1:** Baseline data and results for the study participants.

ID	Age	Gender	UPDRS motor score	MMSE score	Years since diagnosis	PD dominance	Dominant hand	Medication	Switch pattern*
01	79	M	13	29	2	LT	RT	Carbidopa-Levodopa	PH
02	72	M	15	27	1	None	RT	Rasagiline, Carbidopa-Levodopa	PH
03	68	M	12	28	2	RT	RT	Not on medications by choice	RH
04	59	F	24	30	3	LT	RT	Rasagiline, Carbidopa-Levodopa Ropinirole	PH
05	76	M	12	30	3	LT	RT	Carbidopa-Levodopa	RH
06	78	M	11	27	5	RT	LT	Carbidopa-Levodopa	RH
07	71	F	16	30	8	LT	RT	Rasagiline, Carbidopa-Levodopa	RH
08	69	F	11	29	2	LT	RT	Rasagiline	RH
09	54	M	9	28	2	LT	RT	Amantadine, Ropinirole	RH
10	65	M	16	28	5	RT	RT	Amantadine, Rasagiline, Carbidopa-Levodopa, Ropinirole	PH
11	63	M	9	28	1	None	RT	Rasagiline, Ropinirole	RH
12	70	F	21	30	2	None	LT	Rasagiline, Carbidopa-Levodopa	RH
13	70	M	13	29	0	LT	RT	Rasagiline, Procyclidine	EQ
14	66	M	15	29	3	RT	RT	Carbidopa-Lavodopa-Entacapone	RH
15	51	M	13	27	1	None	RT	Amantadine, Rasagiline, Carbidopa-Levodopa	RH
16	53	F	22	29	7	LT	RT	Carbidopa-Lavodopa-Entacapone	RH
17	67	M	26	30	2	LT	RT	Rasagiline, Carbidopa-Levodopa	PH
18	70	M	39	28	1	RT	RT	Amantadine	PH
19	66	F	18	30	3	RT	RT	Rasagiline, Carbidopa-Levodopa	PH
20	53	M	27	29	1	LT	RT	Rasagiline, Ropinirole	RH
21	64	M	49	29	8	Both	RT	Amantadine, Rasagiline, Carbidopa-Levodopa- Entacapone, Biperiden	RH
22	81	M	14	29	1	RT	RT	Carbidopa-Levodopa	PH
23	59	M	9	25	4	LT	RT	Rasagiline, Carbidopa-Levodopa, Carbidopa-Levodopa- Entacapone	PH
24	76	M	46	27	2	RT	RT	Amantadine, Rasagiline, Carbidopa-Levodopa- Entacapone	RH
25	70	F	24	26	5	RT	RT	Amantadine, Rasagiline, Carbidopa-Levodopa, Ropinirole	PH

**Fig 3:**
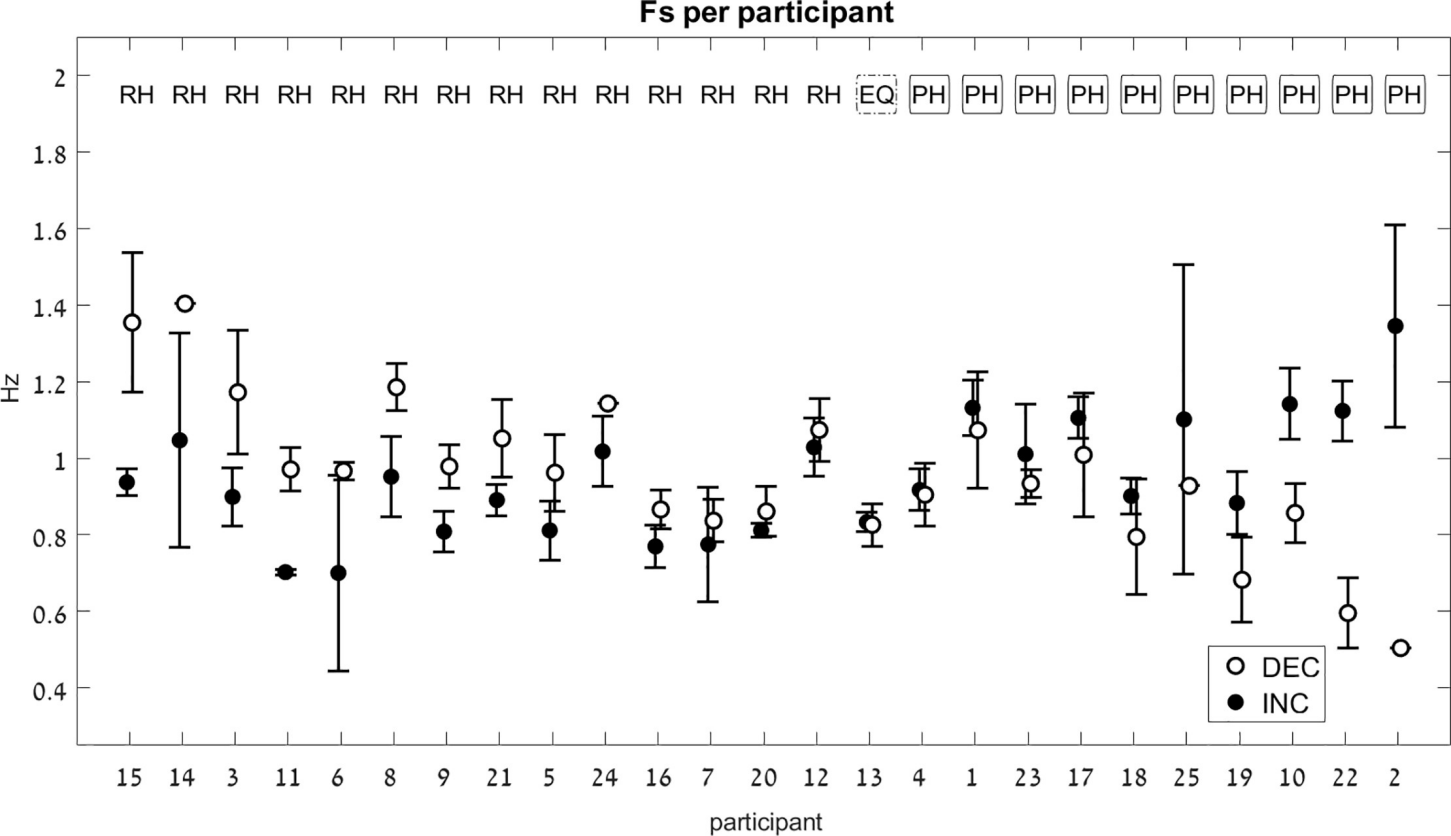
Switch-point frequency (Fs). Average values for Fs on the decreasing-frequency trials (DEC) are marked by a white circle, and those on the increasing-frequency trials (INC) are marked by a black circle. For each pair of INC-DEC results, two letters marked at the top indicate whether this pair shows a reverse-hysteresis pattern (**RH**), a positive hysteresis pattern (**PH**), or the switch point frequency is equal at the INC and the DEC trials (**EQ**). For the sake of clarity, the results are grouped by pattern (RH/EQ/PH), with the participant number on the x-axis corresponding to the chronological order in which participants were tested. Error bars represent standard error.

* The abbreviations here correspond to those in Fig 3: RH indicates a reverse hysteresis, PH indicates a positive hysteresis, and EQ implies there was effectively no difference between the FsINC and FsDEC.

In addition to the 25 participants who completed the experimental session, four participants did not complete it. Of these, two did not complete the session due to self-reported worsening of their PD symptoms during the testing (mostly tremor); one reported not being able to complete the session due to fibromyalgia-induced pain; and one reported feeling discomfort and asked to stop the session.

**Fig 4:**
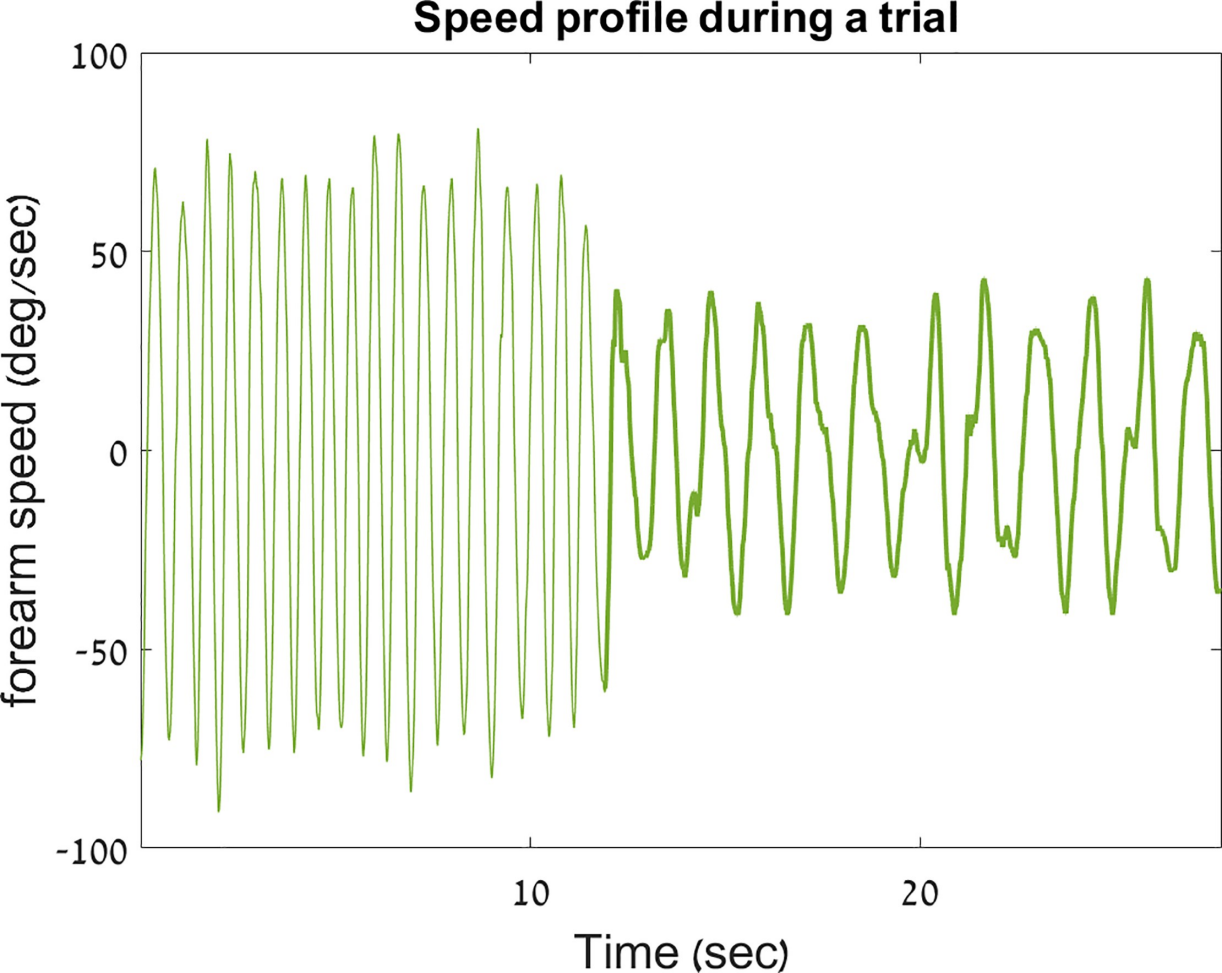
Sample velocity profile on an decreasing-frequency (DEC) trial. Shown here is the velocity profile from an decreasing-frequency trial of participant # 21, the only participant in this study who had a confirmed diagnosis of FOG. A thicker line is used to outline the discrete portion of the trial. This graph demonstrates the percipitous switching that occurs between the rhythmic and the discrete movement types during a trial (shown here is a 30-sec excerpt from a 66.5-sec trial).

The participants performed about seven practice trials, on average, during the training phase. The velocity profile from one participant (#21) performing a DEC trial–is shown in Fig 4.

### No-switch (NS) trials

Twenty-six out of the 199 trials that were recorded, did not contain a switch point. In those trials, participants kept performing the same movement type (either discrete or rhythmic) during the entire trial, never switching to the other type. These were performed by 11 of the participants; of these, nine were INC trials, and 17 were DEC trials.

### Switch frequency (Fs)

For each participant, the average FsDEC and FsINC were calculated from all trials which had a switch point, and the switching pattern categorized as either reverse hysteresis (FsINC < FsDEC, noted as “RH”), positive hysteresis (FsINC > FsDEC, noted as “PH”), or no hysteresis (FsINC = FsDEC, noted as “equal”, or EQ, if the difference between FsINC and FsDEC was 0.01 Hz or less). Fifty-six percent of the participants had a reverse-hysteresis switch pattern. The switch frequencies are shown in Fig 3.

### Predictive control

The data from the 21 participants who had at least two trials with switch points on both INC and DEC trials were included in the statistical analysis.

There was no significant difference between the FsINC (0.9 Hz ± 0.2) and the FsDEC (1.0 Hz ± 0.2) values (p = 0.3; 95% Confidence interval for the difference, using the Hodges-Lehmann estimator: [-0.0668 0.1330]).

## Discussion

Our goals in the current study were to test: (1) whether patients with PD, taking their regular medication, are able to switch between two movement plans of the upper limb when the requirement to switch is implicit; and if so, (2) whether the switch occurs pre-emptively. We asked participants to perform a task that required an implicit (unaware) switch between two movement types. That switch can be made predictively or reactively. We found that more than half of the participants (14 out of 25, 56%) pre-acted, or predictively switched between the two movement types. This was manifest in a reverse-hysteresis switching pattern (RH). These results refute our first hypothesis that patients with PD, who are on medication, have an impaired ability to switch between implicitly specified tasks; They also refute our second hypothesis, that patients with PD will have a difficulty switching *predictively* between the two movement plans.

These results are surprising, since *age* has been documented to have an effect on predictive control of movement and on task switching [9]. It could be expected, then, that the presence of PD, at an older age, would compound the effect, leading to an even more impaired ability to switch between implicitly specified tasks. This is not what we found here. A reduction in predictive control of movement with age has been previously reported [50]. It has been suggested that older adults have an inertial tendency [51] to keep to the same movement plan without changing it, due to a higher cost of switching [52] between tasks, compared to young adults [53]. People with PD have been documented to have a particular difficulty switching between explicit motor tasks while using dopaminergic medications [17,37,38]. The current study design was intended to test whether patients with PD, who are on medication, experience difficulty when switching between *implicitly* specified tasks. We found that patients with PD, who are on medication, switch between implicitly specified tasks at higher rates than healthy older adults do.

We found that 56% of the participants with PD were successful in predictively controlling their movements, showing a reverse-hysteresis switching pattern, compared with 40% reported in a previous study for healthy older adults [9]. It should be noted that in that study, participants were slightly older, on average, than participants in the current experiment (about 71 years old on average, compared with 67 years old, on average, in the current study), and the majority were female participants, whereas here, the majority of the participants were male. It is possible that both these factors–age and gender–affect the performance on the task.

An additional possible explanation for the increased percentages of individuals who pre-acted (56%), compared to that in healthy old adults of a similar age (40%) may be the result of the medication the patients were on. It is possible that the dopaminergic medications afford an advantage in predictively controlling movement, as they help alleviate FOG [6], for example, suggesting a role of dopamine in task switching. Indeed, it has been previously shown that dopamine plays a role in task switching among young and old adults [54]. It has also been found that dopaminergic medications are related to better task switching in patients with PD [55]. In fact, there is evidence to suggest that patients on L-Dopa display impulsive behavior and delay aversion, or “lower switch costs” than when they are off medication [56].

These findings can be explained by the “dopamine overdose” hypothesis which suggests that dopaminergic medications can improve certain cognitive functions, such as the ones responsible for task-switching [17,55,57]. Thus, while patients with PD, who are on medication, may benefit from the advantage of dopamine availability, healthy old adults were found to have a reduction in dopaminergic activity [58]. Studies with contrasting results, on task switching in PD, that have found difficulties in explicit switching between tasks even when patients were on medication [17,37,38], suggest the possibility that the medication selectively improves the ability to implicitly switch between motor tasks. As the beneficial effects of L-Dopa might be dependent on task demands and basal dopamine levels [59], the results reported here can serve as the basis for further exploration of whether PD medication selectively improves *implicit* task switching.

Another factor that may contribute to the relatively high rates of predictively controlling movement within the PD group is selection bias: most patients who chose to participate in our study present with relatively mild PD symptoms (MDS-UPDRS motor score 19±11), and do not suffer from a cognitive decline, as evidenced by the MMSE scores (28±1), and thus may not represent the entirety of the PD population.

The finding that, of the 26 trials that contained no switch point, about two thirds (17/26) were DEC trials, suggests that switching from a rhythmic to a discrete movement type was more challenging for people with PD than the reverse switch (a similar finding was reported for healthy old adults in [9]).

As can be seen in Table 1, there was no correspondence between the switching pattern (PH or RH) and the motor UPDRS score, or the years since diagnosis.

The results we report here suggest that people with PD on medication are able to predictively switch between implicitly specified movement plans of the upper limb. Future research should examine how anticipatory ability of the upper limb correlates with that of the lower limb in people with PD, e.g., through the study of APAs [27–30].

### Predictive control of movement

We interpret the early switching between the two motor tasks (the reverse-hysteresis pattern) as indicating people are controlling their movements predictively [9,32]. The results show that participants performed a mix of classical-hysteresis (positive hysteresis) and reverse-hysteresis switching patterns. This mix has been previously documented in young and old healthy adults performing this task [9], and in young individuals when performing walk-to-run (WR) and run-to-walk (RW) transitions in the lower limb [60–63]. Supporting our predictive-control hypothesis, [63], studying movement-pattern transitions in the lower limb, also interpreted the reverse hysteresis pattern to reflect participants’ *intention*, rather than a switch that is based solely on physical considerations. Thus, data from both the upper and the lower limb are consistent with an interpretation that people engage in predictive control of movement, by changing their movement plan in preparation for an anticipated change in the task requirements. One advantage of pre-action is that it bypasses the inherent delays in feedback-based reactions to a changed context. Indeed, performing an action predictively improves the speed, the accuracy [64,65] and the efficiency [66] of the movement.

Further evidence for pre-action comes from a series of behavioral experiments demonstrating the propensity of participants to start a task early, even at the expense of extra physical effort [67]. The authors termed this unexpected behavior “pre-crastination”, and have since replicated this finding in a variety of task conditions, including across animal species (for a review, see [68]). Yet another group of researchers has found another form of pre-action which they have termed “the mere urgency effect” [69]. It is the tendency to prefer starting a task earlier, even at the cost of a lower reward. These are all examples of making an early change to one’s plans in anticipation of future demands.

Results from young individuals performing the implicit switching task [9] as well as from studies of pre-crastination (e.g., [70,71]) and of predictive control of saccadic eye movements [72] suggest that there is a cognitive component to the predictive control of movement. The cognitive decline reported to be associated with PD [4] would suggest we should find less predictive control in the PD population, but that is not the case, potentially due to the dopaminergic medication they take, as suggested above. It is, however, possible that the findings we report here may not apply to patients at more advanced stages of PD, who may show different switching patterns. This should be tested in future work. Further studies should also be conducted to test the range of tasks in which patients with PD are able to predictively switch between movement plans, and the specific role of dopaminergic medication in facilitating this switching ability.

### Study limitations

A possible limitation of our work is the sample size– 25 individuals with PD. While it is a large sample size compared to previous works with individuals with PD, a larger sample size may be more representative of the population of people with PD. In addition, the participant pool comprised mostly people with PD who present relatively mild manifestations of the disease. It is possible that people at more advanced stages of PD would show a different pattern of switching. Lastly, this study was conducted with people with PD, who were on medication. It would be instructive to conduct this study with a group of patients who are off medication, to uncover the role of dopaminergic medication on switching ability and predictive control.

## Supporting information

S1 Table Rigidity scores from the MDS-UPDRS examination of each of the 25 study participants. *Abbreviations: RUE–Right Upper Extremity; LUE–Left Upper Extremity; RLE–Right Lower Extremity; LLE–Left Lower Extremity.
(DOCX)

S2 Table Medication dosages for each of the 25 study participants. (DOCX)
