# Supplementary material for: Implicit task switching in Parkinson’s disease is preserved when on medication
Source: PLoS One. 2020 Jan 14;15(1):e0227555. doi: 10.1371/journal.pone.0227555 (PMC6959575; doi:10.1371/journal.pone.0227555)
Supplement: S1 Table — *Abbreviations: RUE–Right Upper Extremity; LUE–Left Upper Extremity; RLE–Right Lower Extremity; LLE–Left Lower Extremity. (DOCX) [file pone.0227555.s001.docx]

| **ID** | **Rigidity score** |
| --- | --- |
| 01 | RUE: +1, LUE: +2, LLE: +2 |
| 02 | RUE: +1, LUE: +2 |
| 03 | RUE: +2. LUE: +1 |
| 04 | RUE: +2, LUE: +2 |
| 05 | RUE: +1 |
| 06 | RUE: +2 |
| 07 | RUE: +1, LUE: +1 |
| 08 | RUE: +1, LUE: +2 |
| 09 | RUE: +1, LUE: +2 |
| 10 | RUE: +2, LUE: +2, RLE: +1 |
| 11 | RUE: +1, LUE: +1 |
| 12 | RUE: +2, LUE: +2 |
| 13 | RUE: +1, LUE: +1 |
| 14 | RUE: +2, LUE: +2 |
| 15 | RUE: +2, LUE: +3 |
| 16 | RUE: +3, LUE: +3, Neck +2 |
| 17 | RUE: +2, LUE: +3 |
| 18 | RUE: +2, Neck: +1 |
| 19 | RUE: +3, LUE: +2 |
| 20 | RUE: +1, LUE: +1 |
| 21 | RUE: +1 |
| 22 | RUE: +1 |
| 23 | RUE: +1, LUE: +2, LLE: +1 |
| 24 | RUE: +2, LUE: +2, RLE: +1, LLE: +1 |
| 25 | RUE: +2, LUE: +1, RLE: +1 |
