# Supplementary material for: Implicit task switching in Parkinson’s disease is preserved when on medication
Source: PLoS One. 2020 Jan 14;15(1):e0227555. doi: 10.1371/journal.pone.0227555 (PMC6959575; doi:10.1371/journal.pone.0227555)
Supplement: S2 Table — (DOCX) [file pone.0227555.s002.docx]

| **ID** | **Medication** | **Dosage (for 24 hours)** |
| --- | --- | --- |
| 01 | Carbidopa-Levodopa | 187.5 mg |
| 02 | Carbidopa-Levodopa | 300 mg |
|  | Rasagiline | 1 mg |
| 03 | Not on medications by choice | None |
| 04 | Carbidopa-Levodopa | 750 mg |
|  | Rasagiline, | 1 mg |
|  | Ropinirole | 4 mg |
| 05 | Carbidopa-Levodopa | 200 mg |
| 06 | Carbidopa-Levodopa | 300 mg |
| 07 | Carbidopa-Levodopa | 200 mg |
|  | Rasagiline | 1 mg |
| 08 | Rasagiline | 1 mg |
| 09 | Amantadine | 200 mg |
|  | Ropinirole | 8 mg |
| 10 | Carbidopa-Levodopa | 750 mg |
|  | Amantadine | 200 mg |
|  | Rasagiline | 1 mg |
|  | Ropinirole | 10 mg |
| 11 | Rasagiline | 1 mg |
|  | Ropinirole | 8 mg |
| 12 | Carbidopa-Levodopa | 300 mg |
|  | Rasagiline | 1 mg |
| 13 | Rasagiline | 1 mg |
|  | Procyclidine | 5 mg |
| 14 | Carbidopa-Lavodopa-Entacapone | 400 mg |
| 15 | Carbidopa-Levodopa | 250 mg |
|  | Rasagiline | 1 mg |
|  | Amantadine | 200 mg |
| 16 | Carbidopa-Lavodopa-Entacapone | 300 mg |
| 17 | Carbidopa-Levodopa | 125 mg |
|  | Rasagiline | 1 mg |
| 18 | Amantadine | 200 mg |
| 19 | Carbidopa-Levodopa | 250 mg |
|  | Rasagiline | 1 mg |
| 20 | Rasagiline | 1 mg |
|  | Ropinirole | 8 mg |
| 21 | Carbidopa-Levodopa- Entacapone | 500 mg |
|  | Amantadine | 200 mg |
|  | Rasagiline | 1 mg |
|  | Biperiden | 2 mg |
| 22 | Carbidopa-Levodopa | 500 mg |
| 23 | Carbidopa-Levodopa- Entacapone | 400 mg |
|  | Carbidopa-Levodopa | 200 mg |
|  | Rasagiline | 1 mg |
| 24 | Carbidopa-Levodopa- Entacapone | 400 mg |
|  | Amantadine | 200 mg |
|  | Rasagiline | 1 mg |
| 25 | Carbidopa-Levodopa | 187.5 mg |
|  | Amantadine | 200 mg |
|  | Rasagiline | 1 mg |
|  | Ropinirole | 8 mg |
